# Individual differences and health in chronic pain: are sex-differences relevant?

**DOI:** 10.1186/s12955-019-1182-1

**Published:** 2019-07-22

**Authors:** C. Suso-Ribera, V. Martínez-Borba, R. Martín-Brufau, S. Suso-Vergara, A. García-Palacios

**Affiliations:** 1 Universitat Jaume I, Avda. Vicent Sos Baynat s/n. 12071, Castellón de la Plana, Spain; 2 Universidad de Murcia, Murcia, Spain; 3 Hospital Clinic, Barcelona, Spain; 4 CIBER of Physiopathology of Obesity and Nutrition CIBERobn, CB06/03, Instituto de Salud Carlos III, Barcelona, Spain

**Keywords:** Moderation analyses, Psychological variables, Chronic pain, Health status, Sex differences

## Abstract

**Background:**

Because psychological variables are known to intercorrelate, the goal of this investigation was to compare the unique association between several well-established psychological constructs in pain research and pain-related outcomes. Sex differences are considered because pain is experienced differently across sex groups.

**Methods:**

Participants were 456 consecutive chronic pain patients attending a tertiary pain clinic (mean age = 58.4 years, SD = 14.8, 63.6% women). The study design was cross-sectional. Psychological constructs included personality (NEO-Five Factor Inventory), irrational thinking (General Attitudes and Beliefs Scale), and coping (Social Problem Solving Inventory). Outcomes were pain severity and interference (Brief Pain Inventory) and physical, general, and mental health status (Short Form-36). To decide whether the bivariate analyses and the two-block, multivariate linear regressions for each study outcome (block 1 = age, sex, and pain severity; block 2 = psychological variables) should be conducted with the whole sample or split by sex, we first explored whether sex moderated the relationship between psychological variables and outcomes. An alpha level of 0.001 was set to reduce the risk of type I errors due to multiple comparisons.

**Results:**

The moderation analyses indicated no sex differences in the association between psychological variables and study outcomes (all interaction terms *p* > .05). Thus, further analyses were calculated with the whole sample. Specifically, the bivariate analyses revealed that psychological constructs were intercorrelated in the expected direction and mostly correlated with mental health and overall perceived health status. In the regressions, when controlling for age, sex, and pain severity, psychological factors as a block significantly increased the explained variance of physical functioning (ΔR2 = .037, *p* < .001), general health (ΔR2 = .138, *p* < .001), and mental health (ΔR2 = .362, *p* < .001). However, unique associations were only obtained for mental health and neuroticism (β = − 0.30, *p* < .001) and a negative problem orientation (β = − 0.26, *p* < .001).

**Conclusions:**

There is redundancy in the relationship between psychological variables and pain-related outcomes and the strength of this association is highest for mental health status. The association between psychological characteristics and health outcomes was comparable for men and women, which suggests that the same therapeutic targets could be selected in psychological interventions of pain patients irrespective of sex.

## Background

Chronic pain is defined as a recurrent of persistent pain that lasts longer than 3 months [1]. Current estimates of this disease range from 20 to 30% globally [2, 3] and this disease has become one the leading causes of sick leave and physical disability both in the United States of America and Europe [4, 5]. Unfortunately, projections of future prevalence of this disease are not more encouraging. As life expectancy rises, we are experiencing a change in the age distribution of our populations towards the elderly [6]. Because the prevalence of this disease increases to up to 73.5% for people over 65 years old [7], the ageing of the population is likely to have important implications for chronic pain. Due to its high prevalence, the annual costs of chronic pain has been estimated to exceed those of other major chronic diseases, such as heart problems, cancer, and diabetes [5]. In addition to these economic consequences of the disease, the onset and chronification of pain has an important impact on the quality of life of individuals, with more than 30% of patients in specialized centres meeting criteria for severe depression [8]. As a consequence of the above, chronic pain has become a major health challenge for our societies worldwide and research is needed to improve its current management [9].

Traditionally, pain had been considered to be a sensory experience in which the amount of tissue damage was proportional to the severity of the disease. However, decades of research have evidenced that chronic pain can only be understood within a biopsychosocial framework [10]. Consequently, pain is now defined as a multidimensional “distressing experience associated with actual or potential tissue damage with sensory, emotional, cognitive, and social components” [11].

As a result of this new approach to pain, the inclusion of psychological factors is now frequent in the pain literature [12–14] and psychological treatment has increasingly gained ground as a major non-pharmacological treatment for chronic pain [15–17]. While this has contributed to a better understanding of the disease, a limitation of existing research is that an overwhelming array of psychological factors associated with pain is now available, but the specific, independent contribution of each psychological factor remains unclear. This is important because psychological variables tend to correlate with each other [18, 19], so this overlap between constructs might be leading to redundancy or misleading decisions in current treatments and psychological models of pain.

Thus, the goal of the present study is to explore the unique association of a number of well-established psychological factors in the pain literature, namely personality, coping, and maladaptive forms of thinking, such as catastrophizing and self-criticism [12, 20, 21], in relation so several pain- and health-related outcomes in a sample of chronic pain patients. Importantly, the study will account for sex differences. This is essential because both the experience of pain and health status, as well as the role of psychological factors appear to be sensitive to sex. For instance, women appear to be more sensitive to pain and display more negative responses to it, engage in pain behaviour for longer periods of time, and are more likely to focus on the negative emotions associated with pain, arguably due to higher levels of catastrophizing and neuroticism [22–25], as well as biological sex differences related to the immune system [26–28].

Despite the aforementioned sex differences revealed in the pain literature, the extent to which treatments and mechanisms of change differ for men and women remains unclear [29]. In fact, popular pain models like the Fear Avoidance Model of pain or the Psychological Flexibility Model of pain [30, 31] still tend to ignore sex as a contributor to the pain experience. By comparing the unique association between important psychological factors and the pain experience while considering sex differences, we aim at shedding some light in this regard. Specifically, we expect to find important communalities (i.e., correlations) between the psychological constructs included in the study (i.e., personality, coping, and beliefs like catastrophizing and self-criticism), so that unique associations will be rare. Additionally, we hypothesize that women will present a different psychological profile than men (i.e., higher neuroticism, catastrophizing, and negative coping), which will help understand higher mental distress and higher physical disability in this population. Finally, we expect to find sex differences when exploring the unique association between psychological variables and study outcomes, so that stronger relationships will be revealed in women.

## Methods

### Procedure

Participants were consecutive patients attending the Pain Clinic of the Vall d’Hebron University Hospital in Barcelona (Spain), which is a public, tertiary referral hospital with an area of influence of more than 400,000 residents. Patients were contacted by letter a month before their first medical appointment. The letter included the evaluation protocol together with an explanation of the study goal, its procedures and associated risks, as well as the contact information of the lead researcher, C.S.R. All participants completed the informed consent and questionnaires at home and returned the documents to their doctor the day of their medical appointment.

### Participants

Over a period of 4 years (from early 2012 to late 2015) we contacted 2127 individuals. Of these, 513 patients (24.1% of the contacted patients) agreed to participate and completed the protocol. Difficulties in writing or reading, together with the high number of questions included in the protocol were cited as main reasons for not participating. Further information on participants who declined to participate at this stage could not be collected.

In addition to this, the protocol was not complete for 57 of the 513 patients (11.1%). To facilitate the readability of the text and to minimize the risk of response bias due to small variations in each analysis as a result of different samples, only those individuals with complete information for all variables (*n* = 456) will be used in the analysis. To discuss the generalizability of findings and possible biases, we will compare their characteristics, when available, with those of the non-completers.

### Measures

#### Personality

Several models of personality exist, but the Five Factor Model (FFM) of personality is the most integrative and widely used model in different settings [32], including chronic pain [14]. The FFM describes five bipolar dimensions, which are neuroticism, extraversion, openness to experience, agreeableness, and conscientiousness. High scores in neuroticism are associated with a tendency to experience negative emotions such as anxiety, depression and anger or worry. Extroverts tend to be active, talkative, enthusiast and energetic. Openness to experience defines individuals with a great variety of interests, who tend to be curious, original and sensitive to art. High scores in agreeableness are obtained by altruist, careful and cooperative individuals. Finally, conscientiousness refers to achievement, diligence and hard-work in the positive pole [33].

The FFM of personality was measured with the NEO-Five-Factor-Inventory (NEO-FFI; [33, 34]). In the NEO-FII, individuals are asked to rate the degree to which they agree with 60 statements (12 per scale) using a 5-point Likert scale ranging from 0 = “totally disagree” to 4 = “completely agree.” Scores for each scale range from 0 to 48. The NEO-FFI has adequate psychometric properties (0.66 < α < 0.81; [34]). The internal consistency in our sample was also good (0.69 < α < 0.84).

#### Maladaptive beliefs

The General Attitudes and Beliefs Scale (GABS; [35]) is considered to be the gold standard measure of maladaptive beliefs, as it assesses cognition rather than behaviour [36] and considers the four irrational forms of thinking proposed by Albert Ellis [37, 38]. These are demandingness (i.e., inflexible requirements expressed in terms of ‘have to’, ‘musts’, ‘shoulds’, or ‘oughts’), catastrophizing (i.e., anticipating, focusing, and ruminating about the most negative consequences of an event), low frustration tolerance (i.e., evaluating certain circumstances as unbearable), and self-downing (i.e., a tendency to be excessively critical with oneself.

The present study used a shortened version of the GABS [21], the GABS-SV, which has 24 irrational items (6 for each scale). Participants are asked to rate their degree of agreement using a 5-point Likert-type scale ranging from 0 = “strongly disagree” to 4 = “strongly agree”, so scale score range is 0–24. A reduced version of the GABS has been previously used [39], where its scales showed moderate to good levels of consistency (0.59 < α < 0.70). Internal consistency estimates in our sample were adequate (0.67 < α < 0.86).

#### Coping

The Social Problem solving Inventory – Revised (SPSI-R; [40]) assesses the way individuals cope with real-life problems. It differentiates two components of coping: problem orientation and specific strategies used when solving problems. The former reflect the person’s appraisal of problems and differentiate a positive orientation (i.e., a view of problems as achievable challenges) from a negative orientation (i.e., appraisal of problems as unsolvable situations). Problem orientations are argued to lead to the use of certain strategies, namely planning and solution-testing (i.e., rational problem solving, procrastination (i.e., avoidance style), or rushed behaviour (i.e., impulsive-careless style; [41, 42]).

The short version of the SPSI-R has 25 items, 5 for each scale. Participants are asked to rate their degree of agreement on a 5-point Likert scale, where 0 means “totally disagree” and 4 “completely agree”. Therefore, scores for each scale can range from 0 to 20. All five scales of the short version of the questionnaire have been reported to have a good internal consistency (0.73 > α > 0.86; [43]), which is congruent with scores in our sample (0.68 > α > 0.82).

#### Health

Physical, general, and mental health status were assessed with the Short Form-36 Health Survey [44, 45]. In the questionnaire, physical aspects of health include the ability to perform daily activities (physical functioning) and work-related activities (role physical), and the average intensity of pain in the last 4 weeks (bodily pain). Some components refer to both physical and mental health status, namely the perception of present and future health (general health), vitality (i.e., the evaluation of personal energy), and social functioning (i.e., the interference of health problems in interpersonal life). Role emotional (i.e., the interference of emotions on functioning), and mental health (i.e., overall perception of psychological well-being) mainly reflect psychological and mental aspects of health [46].

Two composite scores of physical and mental health can be obtained for physical and mental health using the aforementioned subscales, which reduces the number of statistical comparisons [45]. However, the use of the physical functioning and general health scales was preferred in the present study. First, because pain intensity had already been measured with a numerical rating scale, which is a more frequent practice in pain research. Additionally, being unemployed or retired is very frequent in pain samples [47], including the present, which makes the role physical scale less relevant. The mental health component will be used as the measure of perceived mental well-being. All three scales (i.e., physical functioning, general health, and mental health) are standardized to have a 0–100 range. Higher scores indicate better health. The psychometric properties of the questionnaire are good (0.78 < α < 0.94; [44]). The internal consistency was also good in our sample (0.75 < α < 0.92).

#### Pain severity and interference

We assessed current pain severity and interference by means of a numerical rating scale ranging from 0 (“no pain/interference”) to 10 (“worst possible pain/interference”) based on the Brief Pain Inventory [48, 49]. This instrument is widely used in the assessment of chronic pain and has become a standard tool [50]. The SF-36 also includes a scale on pain intensity (bodily pain); however, this only evaluates average pain intensity in the last 4 weeks, which might differ from current pain ratings and both are frequently defined as separate assessment domains [51].

### Data analysis

First, we performed a series of descriptive analyses differentiated by sex on our study variables. In doing so, we calculated their sex differences using Student’s t-tests. Differences between males and females will be expressed as Cohen d’s as study variables have different units. Completers and non-completers will be compared using a Chi-square (for sex) and a Mann-Whitney U-test (for age and pain severity and interference) due to the reduced sample size in the non-completers group and the violation of assumptions for parametric tests. The remaining characteristics could not be compared due to missing information in the non-completers condition.

Next, an analysis of moderation was performed to explore whether heterogeneity of associations between psychological variables and pain and health status existed as a function of sex. Testing an interaction is preferred to direct stratification because in the latter, the lack of association in one sex (or in both) could be the consequence of a reduced power in one or both strata (doi: 10.1016/j.jclinepi.2017.09.012.) [52].

To end, a series of Pearson correlations and multivariate regressions were conducted to explore both the bivariate and multivariate relationship between psychological variables (personality, problem solving, and irrational beliefs) and pain- and health-related factors (pain intensity and interference and physical, general, and mental health). Both in the bivariate and the multivariate analyses, the analyses will only be conducted independently as a function of sex when the previous moderation analyses revealed a significant moderation effect of sex. In all other cases, the whole sample will be used.

The multivariate linear regression analyses were performed to explore a health model using psychological variables and controlling for the role of age, sex, and pain severity. Each pain- and health-related factor was used as a dependent variable (i.e., pain severity and interference, physical functioning, general health, and mental health), while age, sex, pain severity, and psychological measures were the independent variables. In a first block, we included age, sex, and pain severity. Psychological factors were added in the second block. Pain severity was not used as a covariate when pain severity was the outcome.

Because independent variables were expected to be significantly correlated, we calculated multicollinearity problems in all regressions by means of the Variance Inflation Factor (VIF). The typically suggested thresholds of 5 or 10 were used to determine multicollinearity problems [53]. To reduce the risk of type I errors due to the large number of statistical analyses performed and to minimize unimportant associations, the critical alpha level for significance in the study will be set at 0.001.

Some patients only completed part of the evaluation protocol due to its length. The analyses will be conducted with the sample of patients will complete data (*n* = 465). Otherwise, it would be hard to identify whether a response bias was introduced due to small variations in each analysis due to different samples for some variables. To discuss the generalizability of findings, the characteristics of the completers’ sample will be compared against those of the non-completers, when available.

The statistical program IBM SPSS Statistics version 22 was used to perform all data analysis [54].

## Results

### Demographic characteristics of the sample and comparison with non-completers

The completer’s sample included 456 participants (63.6% women). Their mean age was 58.4 years (SD = 14.8, age range = 18–89). Most participants had not completed their secondary education (54.1%) and were not working at the time of assessment (68.1%). More than half of the sample was married or in a relationship (60.7%). Most frequent diagnoses were low back pain (58.6%), neck pain (9.1%), post-surgery pain (5.6%), and osteoarthritis (5.4%).

Completers and non-completers were comparable in terms of sex distribution (χ2 = 0.513, *p* = .474) and pain severity (U = 11,360.5, *p* = .169), but non-completers tended to be older in age (U = 9333.5, *p* < .001, mean age for non-completers = 65.21 years, SD = 14.06).

### Sex differences in psychological factors and health outcomes

Our analyses revealed sex differences in pain intensity and physical functioning only, both in favour of men (Table 1). All differences were between small and medium (d between .30 and .39). Male-to-female differences in pain interference, pain duration, perceived general health, mental health, and psychological characteristics (i.e., personality, coping, and beliefs) were all non-significant at an alpha level of 0.001.

**Table 1:** Sample characteristics and sex differences in study variables

	Women *n* = 292	Men *n* = 173	*t*	*d*
Age	59.85 (14.30)	56.08 (15.78)	2.58	0.25
*Pain characteristics*				
Pain intensity	7.96 (1.59)	7.35 (1.78)	3.70*	0.36
Pain interference	8.18 (1.62)	7.88 (1.78)	1.86	0.18
*Health status*				
Physical functioning	30.55 (23.78)	37.40 (24.56)	− 2.96	0.28
General health	34.19 (19.42)	37.25 (19.97)	−1.63	0.16
Mental health	47.63 (21.05)	55.07 (20.93)	−3.69*	0.35
*Personality*				
Neuroticism	25.33 (8.87)	23.54 (9.09)	2.09	0.20
Extraversion	26.59 (8.11)	25.71 (7.84)	1.15	0.11
Openness	22.95 (6.75)	23.67 (6.72)	−1.11	0.11
Agreeableness	32.24 (5.91)	31.30 (6.29)	1.61	0.15
Conscientiousness	31.24 (7.39)	30.96 (6.93)	0.40	0.04
*Coping*				
Positive orientation	9.46 (3.85)	10.06 (3.93)	−1.62	0.15
Negative orientation	8.07 (4.55)	6.94 (4.95)	2.60	0.24
Rational style	9.11 (4.06)	10.34 (4.24)	−3.11	0.30
Impulsive style	5.80 (4.31)	6.11 (4.41)	−0.75	0.07
Avoidant style	3.40 (4.04)	3.98 (4.24)	−1.46	0.14
*Beliefs*				
Demanding	17.50 (3.80)	17.06 (3.72)	1.22	0.12
Catastrophizing	12.18 (6.27)	11.36 (6.06)	1.38	0.13
Frustration intolerance	12.51 (5.72)	12.02 (5.65)	0.90	0.09
Self-downing	6.87 (5.57)	6.13 (5.06)	1.43	0.14

### Sex differences in the association between psychological factors and pain and health outcomes

As seen in Table 2, none of the interaction terms in moderation analysis indicated a significantly different association between predictors (i.e., psychological variables) and outcomes (i.e., pain and health status) as a function of sex (all *p* values were larger than .001). Consequently, further analyses were not segregated by sex.

**Table 2:** Regression analyses with sex as a moderator of the relationship between psychological factors and study outcomes (whole sample)

	Criterion	PF		GH		MH		PS		PI	
Model	Predictor	*β*	*ΔR* ^2^	*β*	*ΔR* ^2^	*β*	*ΔR* ^2^	*β*	*ΔR* ^2^	*β*	*ΔR* ^2^
1	N	−0.09	.016	− 0.19	.109^*^	− 0.65^*^	.326^*^	0.18	.023^*^	0.26	.050^*^
	Sex	0.12	.013	0.04	<.001	0.10	.008	−0.16^*^	.024^*^	− 0.07	.003
	N^*^Sex	−0.04	<.001	−0.15	.001	0.10	<.001	−0.04	<.001	−0.04	<.001
2	E	0.09	.011	0.13	.043^*^	0.34	.080^*^	−0.22	<.001	−0.32	.005
	Sex	0.14	.019	0.09	.006	0.17^*^	.028^*^	−0.17^*^	.026^*^	−0.09	.007
	E^*^ Sex	0.04	<.001	0.09	<.001	−0.05	<.001	0.23	.004	0.24	.004
3	O	0.06	.021	0.15	.015	.017	.006	−0.14	.001	−0.07	<.001
	Sex	0.13	.014	0.07	.003	0.16	.022	−0.17^*^	.027	−0.09	.003
	O^*^Sex	0.09	<.001	−0.02	<.001	−0.09	<.001	0.10	<.001	0.08	<.001
4	A	0.17	<.001	0.25	<.001	0.31	.009	0.14	.002	−0.09	<.001
	Sex	0.13	.015	0.07	.003	0.17^*^	.026^*^	−0.17^*^	.027	−0.09	.004
	A^*^Sex	−0.21	.003	−0.31	.008	−0.21	.002	−0.09	<.001	0.07	<.001
5	C	0.24	.020	0.09	.044^*^	.023	.076^*^	−0.12	<.001	− 0.21	.001
	Sex	0.14	.018	< 0.01	.004	0.16^*^	.025^*^	−0.17^*^	.026^*^	−0.09	.006
	C^*^Sex	−0.09	<.001	0.55	<.001	0.05	<.001	0.14	<.001	0.16	<.001
6	PPO	0.25	.026^*^	0.12	.059^*^	0.28	.048^*^	−0.17	.001	−0.35	<.001
	Sex	0.13	.013	0.06	.001	0.14	.018	−0.17^*^	.027^*^	−0.09	.004
	PPO^*^Sex	−0.10	<.001	0.13	<.001	−0.07	<.001	0.14	<.001	0.33	.010
7	NPO	−0.26	.041^*^	−0.34	.126^*^	−0.57^*^	.265^*^	0.13	.015	0.19	.025^*^
	Sex	0.12	.010	0.03	<.001	0.09	.006	−0.16^*^	.023^*^	− 0.07	.003
	NPO^*^Sex	0.07	<.001	−0.02	<.001	0.07	<.001	0.03	<.001	−0.04	<.001
8	RPS	0.05	.005	−0.07	.018	0.21	.037^*^	−0.26	.001	−0.23	<.001
	Sex	0.13	.014	0.05	.001	0.13	.015	−0.18^*^	.026^*^	−0.09	.004
	RPS^*^Sex	0.02	<.001	0.22	.003	−0.03	<.001	0.24	.004	0.21	.003
9	AS	−0.30	.034^*^	−0.30	.047^*^	−0.54^*^	.081^*^	0.17	<.001	0.22	<.001
	Sex	0.15	.021^*^	0.09	.006	0.17^*^	.028^*^	−0.17^*^	.026^*^	− 0.09	.005
	AS^*^Sex	0.10	<.001	0.08	<.001	0.25	.005	−0.17	.001	−0.18	.002
10	ICS	−0.32	.020	−0.21	.033^*^	−0.35	.054^*^	0.36	.018	0.21	.008
	Sex	0.14	.018	0.08	.005	0.16^*^	.024^*^	−0.18^*^	.030^*^	−0.09	.006
	ICS^*^Sex	0.18	.002	0.02	<.001	0.12	<.001	−0.23	.004	−0.12	<.001
11	DEM	−0.25	.006	−0.13	.012	−0.33	.041^*^	0.17	.002	0.17	.002
	Sex	0.13	.015	0.07	.002	.015	.019	−0.17^*^	.029^*^	−0.09	.007
	Sex^*^DEM	0.18	.002	0.02	<.001	0.14	<.001	−0.12	<.001	−0.13	.002
12	CAT	−0.15	.013	−0.20	.073^*^	−0.57^*^	.219^*^	0.13	<.001	0.11	.011
	Sex	0.13	.014	0.06	.002	0.13	.013	−0.17^*^	.027^*^	−0.08	.004
	CAT^*^Sex	0.03	<.001	−0.08	<.001	0.12	<.001	−0.10	<.001	−0.01	<.001
13	LFT	−0.06	.006	−0.12	.086^*^	−0.59^*^	.227^*^	−0.03	.001	0.05	.006
	Sex	0.13	.016	0.06	.002	0.13	.016	−0.17^*^	.027^*^	−0.08	.005
	LFT^*^Sex	−0.03	<.001	−0.19	.002	0.13	<.001	0.08	<.001	0.04	<.001
14	SD	0.10	.017	−0.11	.096^*^	− 0.45^*^	.212^*^	0.06	.004	0.17	.024^*^
	Sex	0.13	.014	0.05	.001	0.12	.012	−0.17^*^	.026^*^	−0.08	.003
	SD^*^Sex	−0.24	.005	−0.21	.003	< 0.01	<.001	< 0.01	<.001	−0.01	<.001

*β* = standardized beta from the last step of the regression equation. Sex (1 = female; 2 = male). All predictors were centered

*N* neuroticism, *E* extraversion, *O* openness to experience, *A* agreeableness, *C* conscientiousness, *PPO* a positive problem orientation, *NPO* a negative problem orientation, *RPS* rational problem solving, *ICS* impulsive-careless style, *AS* avoidant style, *DEM* demandingness, *CAT* catastrophizing, *LFT* low frustration tolerance, *SD* self-downing, *PF* physical functioning, *GH* general health, *MH* mental health, *PS* pain severity, *PI* pain interference

*p < .001

### Bivariate associations

Pearson correlations were calculated altogether for men and women (Table 3). Results revealed that several psychological constructs were significantly intercorrelated. Overall, these correlations were between weak and moderate. In general, we found extraversion and conscientiousness to be positively intercorrelated and significantly associated with so-called positive psychological constructs (i.e., a positive problem orientation and rational problem solving), while negatively linked with arguably negative psychological variables (i.e., neuroticism, a negative problem orientation, impulsive-careless style, avoidant style, and all forms of irrational thinking except for demandingness). The opposite direction of associations was revealed for neuroticism.

**Table 3:** Bivariate associations between study variables (whole sample)

	Personality				Coping					Beliefs				Health & Pain status				
	E	O	A	C	PPO	NPO	RPS	ICS	AS	DEM	CAT	LFT	SD	PF	GH	MH	PS	PI
N	−.40^*^	−.07	−.26^*^	−.41^*^	−.30^*^	.60^*^	−.20^*^	.32^*^	.34^*^	.26^*^	.54^*^	.56^*^	.54^*^	−.14	−.33^*^	−.59^*^	.16^*^	.23^*^
E		.26^*^	.23^*^	.41^*^	.36^*^	−.34^*^	.23^*^	−.08	−.25^*^	−.11	−.24^*^	−.28^*^	−.28^*^	.12	.21^*^	.32^*^	.01	−.09
O			.02	.08	.22^*^	−.13	.26^*^	−.04	−.11	.01	−.05	−.04	.02	.15	13	.13	−.05	−.01
A				.23^*^	.11	−.03	.05	−.09	−.10	.02	−.11	−.11	−.15	−.04	−.04	.16^*^	.07	−.02
C					.54^*^	−.41^*^	.51^*^	−.38^*^	−.42^*^	−.01	−.33^*^	−.35^*^	−.41^*^	.15	.21^*^	.29^*^	.02	−.06
PPO						−.29^*^	.65^*^	−.07	−.24^*^	.02	−.22^*^	−.25^*^	−.32^*^	.17^*^	.25^*^	.26^*^	−.06	−.04
NPO							−.14	.41^*^	.53^*^	.29^*^	.56^*^	.56^*^	.52^*^	−.21^*^	−.36^*^	−.53^*^	.13	.17^*^
RPS								−.18^*^	−.13	.11	−.09	−.15^*^	−.24^*^	.09	.14	.22^*^	−.06	−.05
ICS									.48^*^	.12	.34^*^	.31^*^	.37^*^	−.15	−.19^*^	−.28^*^	.14	.10
AS										.20^*^	.39^*^	.40^*^	.41^*^	−.19^*^	−.22^*^	−.31^*^	−.01	.04
DEM											.53^*^	.49^*^	.19^*^	−.09	−.12	−.22^*^	.07	.05
CAT												.81^*^	.61^*^	−.12	−.28^*^	−.45^*^	.05	.12
LFT													.62^*^	−.09	−.30^*^	−.45^*^	.06	.09
SD														−.14	−.31^*^	−.47^*^	.08	.16^*^
PF															.47^*^	.38^*^	−.44^*^	−.49^*^
GH																.47^*^	−.35^*^	−.36^*^
MCS																	−.26^*^	−.35^*^
PS																		.66^*^

*N* neuroticism, *E* extraversion, *O* openness to experience, *A* agreeableness, *C* conscientiousness, *PPO* a positive problem orientation, *NPO* a negative problem orientation, *RPS* rational problem solving, *ICS* impulsive-careless style, *AS* avoidant style, *DEM* demandingness, *CAT* catastrophizing, *LFT* low frustration tolerance, *SD* self-downing, *PF* physical functioning, *GH* general health, *MCS* mental composite score, *PS* pain severity, *PI* pain interference

^*^p < .001

Table 3 also shows the bivariate associations between psychological variables and study outcomes (i.e., health scales and pain intensity ratings). Overall, psychological factors were more strongly associated with perceived general health status and mental health than with physical functioning, pain severity, and pain interference. Specifically, neuroticism, a negative problem orientation, avoidant coping and impulsive coping, catastrophizing, frustration intolerance, and self-downing correlated to poorer perceived general health status and mental health (−.19 ≤ r ≤ −.57, all *p* < .001), while positive associations with both outcomes were revealed for extraversion, conscientiousness, a positive problem orientation, and rational coping (.16 ≤ r ≤ .30, all *p* < .001). Only openness (r = .15, *p* < .001), a positive (r = .16, *p* < .001) and a negative problem orientation (r = −.19, *p* < .001), and avoidant coping (r = −.18, *p* < .001) were associated with physical functioning, while only neuroticism correlated to pain severity (r = .15, *p* < .001) and interference (r = .22, *p* < .001).

### Unique associations between psychological factors and pain and health outcomes

The multivariate regressions revealed that psychological factors, as a block, added significant variance to the prediction of mental health status (ΔR2 = .352, *p* < .001) and overall perceived health (ΔR2 = .166, *p* < .001) above and beyond age, sex, and pain severity levels (Table 4). Neuroticism (β = − 0.30, *p* < .001) and a negative problem orientation (β = − 0.26, *p* < .001) were the only factors significantly linked to mental health status when controlling for all the covariates and the remaining psychological constructs, while no psychological variable was uniquely and significantly associated with general health status. Psychological variables failed to add significant variance to the prediction of physical functioning, pain severity, and pain interference. Only pain severity was uniquely associated with physical functioning (β = − 0.38, *p* < .001) and pain interference (β = 0.69, *p* < .001).

**Table 4:** Predicting study outcomes from psychological variables (whole sample)

	Criterion	PF		GH		MH		PS		PI	
Block		*ΔR* ^2^	*β*	*ΔR* ^2^	*β*	*ΔR* ^2^	*β*	*ΔR* ^2^	*β*	*ΔR* ^2^	*β*
1	*Covariates*	.278^*^		.123^*^		.080^*^		.051^*^		.443^*^	
	Age		−0.29^*^		−0.08		−0.04		0.17^*^		−0.05
	Sex		−0.05		−0.02		0.07		−0.11		0.04
	Pain severity		−0.39^*^		−0.29^*^		−0.16^*^		*–*		0.68^*^
2	*Psychological variables*	.037^*^		.138^*^		.362^*^		.068		.016	
	*Personality*										
	Neuroticism		< 0.01		−0.11		−0.30^*^		0.22^*^		0.08
	Extraversion		0.02		0.06		0.08		0.05		−0.06
	Openness		0.05		0.05		0.03		−0.01		0.04
	Agreeableness		−0.02		−0.10		0.06		0.06		−0.02
	Conscientiousness		0.12		0.02		−0.07		0.12		−0.02
	*Coping*										
	Positive orientation		0.08		0.11		−0.03		−0.08		0.10
	Negative orientation		−0.01		−0.10		−0.18^*^		0.05		0.04
	Rational style		−0.11		−0.07		0.10		0.01		−0.04
	Impulsive style		0.03		0.01		< 0.01		0.17		−0.08
	Avoidant Style		−0.12		−0.03		−0.03		−0.11		0.01
	*Beliefs*										
	Demanding		0.01		0.05		−0.11		0.04		−0.03
	Catastrophizing		−0.01		0.03		−0.05		−0.14		0.11
	Frustration intolerance		0.05		−0.09		−0.03		0.02		−0.12
	Self-downing		−0.06		−0.11		−0.13		0.04		0.08

*β* = standardized beta from the last step of the regression equation. *R*^2^ change is adjusted

*PF* physical functioning, *GH* general health, *MH* mental health, *PS* pain severity, *PI* pain interference

^*^p < .001

In the regressions, all VIF values were found to be lower than 4, indicating no multicollinearity problems.

## Discussion

The current investigation had a focus on comparing the role of important psychological factors that are known to be associated with the pain experience, taking sex differences into account. As revealed in previous research, results in the present investigation indicated significant associations between psychological variables and pain and health outcomes [10], as well as sex differences in some health characteristics [22, 25]. Unique to the present study is that redundancy exists in the relationship between psychological variables and pain- and health-related outcomes. Most importantly, the association between psychological factors and pain and health outcomes was not sensitive to sex. Research in the past decades has repeatedly supported the multidimensional nature of the pain experience, which is now understood as an interplay between biological, psychological, and social factors [10]. This new approach to pain has resulted in an explosion in the number of psychological factors that have been argued to play a role in the experience of pain. The present study replicated existent findings, supporting the important association between psychological factors and health status in pain patients [55]. On the one hand, we found a number of psychological factors to be negatively related to health status, namely neuroticism, some coping variables (i.e., a negative problem orientation and impulsive-careless and avoidant strategies), and all irrational forms of thinking (i.e., demanding, catastrophizing, low frustration tolerance, and self-downing). On the other hand, associations between psychological factors and positive outcomes were also replicated, thus revealing an arguably positive role of extraversion, conscientiousness, a positive problem orientation, and rational problem-solving in the pain experience.

A contribution of the present investigation to the literature is the analysis of sex differences in the relationship between the aforementioned psychological variables and pain and health outcomes. Contrary to our expectations, the association between psychological factors and outcomes was comparable for men and women. While the literature has revealed biological and psychological differences in the experience of pain between men and women [22–28], our study indicates that these differences are not likely to extend to how psychological factors are associated with pain and health status. In a period when cost-effective interventions are considered an urge [56], a comparable associations between psychological variables and pain and health outcomes for men and women is important as it supports the implementation of mixed, group psychological interventions. In fact, group interventions including both men a women is a frequent practice in chronic pain psychological treatments [57]. The present study findings suggest that segregating psychological interventions as a function of sex would not be justified in terms of how psychological factors are expected to impact on outcomes as a function of sex.

Another strong point of the present study was the comparison of several psychological factors that are important in the pain experience, namely personality, coping, and irrational thinking, to explore their unique association with pain and health status when controlling for the association of the others. As predicted, we found psychological constructs to be intercorrelated, which resulted in a reduced number of unique associations in our regression analyses. This is important because the reduction of key intervention targets to a more manageable set might serve guide psychological treatments in a more effective way (i.e., simplifying treatments to crucial elements). Specifically, our investigation revealed that neuroticism and a negative problem orientation are likely to be relevant factors to understand the mental well-being of pain patients above and beyond other psychological characteristics.

On the one hand, neuroticism has become a matter of public health concern [58] and a goal of the new generation treatments for emotional problems, such as the transdiagnostic treatment for emotional disorders [59, 60]. Our results suggest that this should be the case in chronic pain too. While personality traits are quite stable constructs, there is evidence to suggest that changes in personality occur during the lifespan [61] and some encouraging examples of successful psychological treatments changing personality already exist [62–64], so future studies in chronic pain should address neuroticism as a key factor to be included in psychological treatments for pain.

On the other hand, a negative problem orientation is viewed as narrow and rigid form of coping [65], which is frequently associated with poorer health status [66]. The study of problem solving and problem orientation in pain settings is not new and, decades ago, there was a call for cognitive-behavioural interventions in chronic pain to include problem solving into existent programs [67]. Problem solving has also been tested in other populations, such as patients with personality disorders [68], individuals with severe disabilities [69], and caregivers [70, 71], among others. To date, however, the majority of treatment programs in chronic pain fail to incorporate problem solving [72–75] and existent models of pain behaviour, such as the Fear Avoidance Model of pain or the Psychological Flexibility Model of pain [30, 31], tend to ignore problem solving. The present study suggests that, compared to other psychological variables, a negative problem orientation, together with neuroticism, might be important target outcomes in psychological interventions for chronic pain patients.

An interesting result in the present study was that the contribution of psychological factors was stronger for psychological (i.e., the mental health) or a combination of psychological and physical outcomes (i.e., perceived general health), as opposed to the more physical dimensions of health (i.e., pain intensity and interference and physical functioning). This is a frequently finding in the pain literature and is consistent with studies showing that psychological treatments are more likely to impact positively on psychological well-being than on pain reports [15, 57, 76, 77]. While this might be interpreted as a limitation of psychological treatments, it might as well help guide interventions in a more effective manner (i.e., prioritizing psychological treatment when the goal is to reduce of psychological symptoms as opposed pain levels).

The present study certainly has limitations. Study design was cross-sectional, so the mechanisms underlying the revealed associations remain speculative and causal inferences should be made with caution. For instance, the study of the relationship between physical functioning and psychological constructs as a function of sex is likely to be “a chicken and egg situation”, in which correlational studies cannot shed light on whether sex differences are due to differences in physical functioning, psychological characteristics, or other factors. Additionally, despite we included a number of important psychological constructs that are present both in the pain and the health literature in general, the list is far from complete. A more comprehensive list, however, would require even larger sample sizes, especially if sex-sensitive studies are to be conducted. Despite this, the goal of the present study was not to compare the contribution of all psychological factors that have been investigated in pain settings, but to shed some light on the communalities between psychological factors in the contribution of health outcomes and the important role of sex in such associations. Another study refers to both the specificity of the sample used (i.e., adults with heterogeneous pain characteristics, but mostly musculoskeletal pain) and the fact that a large number of individuals approached by letter (more than 75% of them) were not willing to complete the protocol, mostly because they found it too time-consuming and demanding. This points out to the need to reduce assessment protocols and the importance of developing psychometrically-sound, shortened versions of existing questionnaires that maximize participation rates by reducing burden, specially of older participants (i.e., note that non-completers were found to be older). Other strategies, such as conducting assessments onsite and compensating participants are also frequent procedures, but were not feasible in the present investigation. Finally, it is important to note that comorbidities, which are known to be frequent in chronic pain patients [78], were not investigated in the present investigation. Therefore, sex differences in comorbidities, as well as the association between comorbidities and outcomes and the association of psychological factors with outcomes while controlling for the role of comorbidities could not be investigated.

## Conclusions

To conclude, the present investigation revealed that psychological factors share significant variance in the prediction of outcomes in pain settings, thus supporting a preference for parsimonious and integrative models of health as opposed to complex models that include a wide array of presumably unrelated psychological factors. The list of psychological factors present in the pain literature is now very extensive [79] and it is about time that treatment targets are reduced to a more manageable list. The present study is a step in this direction. Additionally, the present study had a focus on personalizing pain treatments by exploring whether there was preliminary support for a differential association of psychological factors and health outcomes as a function of sex. Different to previous research suggesting that sex differences may be important in the experience of pain [29, 56], our study supports targetting the same psychological factors for both men and women with chronic pain. To the best of our knowledge, there is no research that has tested the effect of adapting psychological treatments for pain according to sex differences. In the light of our findings, segregating by sex might not be a necessary practice in psychological interventions for chronic pain, which is likely to make treatments more cost-effective (i.e., by facilitating the creation of groups irrespective of the sex characteristics of patients seeking treatment).

## Data Availability

Data will be made available upon reasonable request.

## Abbreviations

FFM: Five Factor Model
GABS: General Attitudes and Beliefs Scale
GABS-SV: General attitudes and beliefs scale - short version
NEO-FFI: NEO Five-Factor-Inventory
SPSI-R: Social Problem Solving Inventory - Revised
VIF: Variance Inflation Factor
